# Effects of Chinese Liquors on Cardiovascular Disease Risk Factors in Healthy Young Humans

**DOI:** 10.1100/2012/372143

**Published:** 2012-08-01

**Authors:** Ju-Sheng Zheng, Jing Yang, Tao Huang, Xiao-Jie Hu, Ming Luo, Duo Li

**Affiliations:** ^1^Department of Food Science and Nutrition, Zhejiang University, 866 Yuhangtang Road, Hangzhou 310058, China; ^2^Centre of Nutrition and Food Safety, APCNS, 866 Yuhangtang Road, Hangzhou 310058, China

## Abstract

*Objectives*. To elucidate whether consumption of two Chinese liquors, tea-flavor liquor (TFL) and traditional Chinese liquor (TCL) have protective effects on cardiovascular disease (CVD) risk factors in healthy human subjects. *Methods*. Forty-five healthy subjects (23 men, 22 women), aged 23–28, were recruited and randomized into two groups: TFL and TCL, and consumed 30 mL/day (45% (v/v) alcohol) of either liquor for 28 days. *Results*. Serum high-density lipoprotein cholesterol/low-density lipoprotein cholesterol (HDL-C/LDL-C) and apolipoprotein A1 were significantly increased, and total cholesterol (TC) and TC/HDL-C were significantly decreased after the intervention in both groups (*P* < 0.05). Serum uric acid (*P* = 0.004 for TFL, *P* = 0.001 for TCL), glucose (*P* < 0.001 for TFL, *P* < 0.001 for TCL) and endothelial adhesion molecules (*P* < 0.05) were significantly decreased after the intervention. ADP-induced whole blood platelet aggregation was also significantly decreased after the intervention in both TFL and TCL groups (*P* < 0.05). *Conclusions*. TFL and TCL consumption had protective effects on CVD risk factors in young humans. However, the results were valid only for 28 days, and that the possibility of adverse effect (liver, kidney) of chronic alcohol consumption should be considered.

## 1. Introduction

 Moderate alcohol consumption has been related to decreased risk of cardiovascular disease (CVD) in many epidemiological studies [1–3]. Protective effects of red wine on atherosclerosis were best investigated [4, 5]; however other kinds of alcoholic beverages, such as liquor and beer, were also found to be as beneficial as red wine [6]. Chinese liquor, a special series of distilled spirit, was normally made from sorghum or a mixture of barley, corn, rice, wheat, and sorghum, containing abundant volatile components including esters and organic acids [7], and it was widely consumed in China, having an annual consumption of more than 7 million kiloliters. Nonalcoholic components, such as esters and organic acids in Chinese liquor might also participate in the action to prevent CVD risk factors. However, to our knowledge, there were still no chronic/short-term human intervention studies with regard to the CVD protective effects of Chinese liquors.

Short-term randomized paralleled human trial was conducted in the present study to examine the effects of moderate Chinese liquor consumption on CVD risk factors in healthy young men and women. Two Chinese liquors, traditional Chinese liquor (TCL) and Chinese tea-flavor liquor (TFL), containing the same ethanol content but different total ester and total organic acid concentrations were chosen to observe whether nonalcoholic components and ethanol could exert different synergetic effects on CVD risk factors.

## 2. Materials and Methods

### 2.1. Chinese Liquor Preparation

Two kinds of Chinese liquors: TCL and TFL, were used in this study. Both liquors (45% (v/v) alcohol) were provided by Guizhou Meijiao Co., Ltd., Guizhou, China. TCL was fermented from sorghum, corn, sticky rice, wheat and rice, aged for two years; while green tea was added to the TCL fermentation materials to make TFL, and it was aged for one year. The materials used were firstly cooked and then mixed with Daqu powder (containing various microorganisms including bacteria, yeast, and fungi). The mixed materials were fermented for 70 days under anaerobic conditions in a solid state, distilled out with steam and aged in sealed pottery jars in order to achieve balanced aroma.

Total organic acid and ester of the liquors were analyzed by the method issued by the Chinese National Standardization Committee (no. 10345-2007). Volatile compounds of the liquors were detected using gas chromatographic together with flame ionization detection. A DB-FFAP capillary column (30 m × 0.25 mm id, 0.25 *μ*m film thickness, J&W, USA) was used. The oven temperature was initially set at 50°C for 6 minutes, then increased to 240°C at 4°C/min and held for 5 minutes and nitrogen was used as carrier gas. Butyl acetate was used as the internal standard.

### 2.2. Subjects and Study Design

The study protocol was approved by the Ethics Committee of Department of Food Science of Nutrition, Zhejiang University, China. All participants gave informed consent. Forty-six healthy subjects (23 males, 23 females), aged 23–28, were recruited through an advertisement at Zhejiang University, China. All the participants were Chinese. Exclusion criteria were smokers, participants taking any drug or vitamin/mineral/botanical supplement, participants having a history of hypertension, diabetes mellitus, alcoholic liver disease or CVD. One female withdrew due to personal reasons during the study. Subjects were required to consume TFL or TCL and were randomized into two groups: TFL (*n* = 23; *n* = 12 for men; *n* = 11 for women) and TCL (*n* = 22; *n* = 11 for men; *n* = 11 for women); and all the subjects experienced two weeks of total abstinence from alcohol; tea, coffee and grape consumption were also limited. Then all the subjects consumed 30 mL (45% (v/v) alcohol) of TFL or TCL once per day for 28 days and abstained from other alcohol; tea, coffee and grape consumptions were limited during this period. All the subjects came to a departmental office to drink liquor after lunch or supper once per day. Each subject was required to pursue their usual diet and physical activity; and 3-day diet records before day 0 and day 28 were also required. Dietary energy and nutrient intake of each subject was assessed using the computer program “Diet Analysis” (Cao Aihong, China). All the enrolled subjects underwent anthropometric measurements including height and body weight by an interviewer before and after the intervention.

Blood samples were collected at day 0 and the end of intervention (day 28). All the subjects arrived at the Campus Hospital of Zhejiang University at 8 : 00 am following an overnight fast and relaxed for 10 min. Blood pressure was then measured and venous blood samples were taken; serum were obtained and stored at −70°C for laboratory analysis; whole blood samples were used for whole blood platelet aggregation testing and full blood examination within 4 hours.

### 2.3. Laboratory Analyses

Serum glucose, total cholesterol (TC), triacylglycerol (TG), uric acid, high-density lipoprotein cholesterol (HDL-C), low-density lipoprotein cholesterol (LDL-C), high-sensitivity C-reactive protein (hs-CRP), common liver and kidney function parameters were analyzed on HITACHI 7020 chemistry analyzer using enzyme-based colorimetric test or colorimetric test supplied by Diasys Diagnostic Systems (Shanghai) Co., Ltd. Insulin was measured by ARCHITECT insulin reagent kit (Abbott Laboratories, Abbott Park, IL, USA). Full blood examination was conducted using Mindray BC 5500 Hematology Analyzer (Mindray Bio-Medical Electronics Co., Ltd, Shenzhen, China). Whole blood platelet aggregation (maximal aggregation) was obtained using adenosine diphosphate (ADP) (0.5 *μ*mol/L, 2.0 *μ*mol/L) and epinephrine (2.78 *μ*mol/L, 5.56 *μ*mol/L) as agonists in a two-channel automatic whole blood aggregometer (Chrono-Log 490 Model; Chrono-Log, Havertown, PA).

Apolipoprotein A1 (apoA1) and apolipoprotein B (apoB) were determined by particle-enhanced immunonephelometry; serum intercellular adhesion molecule-1 (ICAM-1) and vascular cell adhesion molecule-1 (VCAM-1) were determined by enzyme-linked immunoabsorbent assay (ELISA, R&D Systems, Minneapolis, MN). Homeostasis Model Assessment (HOMA) score was calculated as Fasting Insulin × Fasting Glucose/22.5 [8].

### 2.4. Statistical Analyses

Two-tailed paired *t*-test was used to compare the changes within the same group. Repeated measure ANOVA was used to evaluate effects of liquor type, time, and the interaction of liquor type and time on tested values. Sample size was chosen based on previous randomized controlled trails of red wine [4, 9]. This sample size gave it 0.80 power (*α* = 0.05, two-sided) to detect a change of 0.46 mmol/L for HDL-C (SD = 0.55) and 0.27 mmol/L for TG (SD = 0.32) in our studies. Values with a skewed distribution (ICAM-1, VCAM-1) were transformed to their ln forms for analyses. The values were reported as mean ± SD for all the tables. Differences of the tested values before and after the intervention were indicated in the tables and figures as mean differences with their 95% confidence intervals (CIs). Differences were considered significant if *P* < 0.05. SPSS version 16.0 (SPSS Inc., Chicago, IL, USA) was used for data analyses.

## 3. Results

### 3.1. Baseline Characteristics, Intervention Compliance, and Dietary Intake

Forty-five subjects were included (mean age 23.5 ± 1.4, mean body mass index (BMI) 21.2 ± 1.9 kg/m^2^). All the included subjects finished the 28-day intervention. The baseline characteristics of all the subjects were shown in Table 1. There were no significant differences in mean daily dietary nutrient and energy intake within and between groups; energy intake of men was much higher than that of women within each intervention group.

### 3.2. Identification of Liquor Composition

Concentrations of total organic acid were 0.22 g/L and 0.83 g/L for TFL and TCL, respectively, and concentrations of total ester were 1.58 g/L and 2.64 g/L for TFL and TCL. TCL had higher total organic acid and ester concentrations than TFL. The most abundant three volatile compounds for TFL were ethyl lactate (0.76 g/L), ethyl hexanoate (0.70 g/L), and isoamylol (0.27 g/L) and were ethyl hexanoate (1.2 g/L), ethyl lactate (0.71 g/L), and acetic acid (0.63 g/L) for TCL.

### 3.3. Changes in Fasting Serum Lipids and Blood Pressure in Young Subjects

Compared with day 0, serum TC (*P* = 0.01), TG (*P* = 0.001), and TC/HDL-C (*P* < 0.001) were significantly decreased, and apoA1 (*P* = 0.004) and HDL-C/LDL-C (*P* = 0.002) significantly increased in TFL group at day 28. Serum TC (*P* = 0.001), LDL-C (*P* = 0.043) and TC/HDL-C (*P* < 0.001) were decreased, and apoA1 (*P* = 0.002) and HDL-C/LDL-C (*P* = 0.004) increased in TCL group at day 28 compared with day 0 (Table 2). Serum HDL-C in females was significantly increased in both TFL (*P* < 0.001) and TCL (*P* = 0.048) group (Table 3).

Diastolic blood pressure (DBP) was significantly increased at day 28 compared with day 0 in TFL group (*P* = 0.001). But systolic blood pressure (SBP) was significantly decreased (*P* = 0.021) at day 28 compared with day 0 in TFL group (Table 2).

### 3.4. Changes in Fasting Serum Glucose, Insulin, and HOMA Score in Young Subjects

Fasting serum glucose levels were significantly decreased in both groups at day 28 compared with day 0; however, insulin levels (*P* = 0.018) were significantly increased in TCL group (Table 2). No significant changes were observed for HOMA score in both groups.

### 3.5. Changes in Fasting Serum Uric Acid and Endothelial Adhesion Molecules in Young Subjects

Serum uric acid was significantly reduced after the intervention compared with day 0 in TFL (*P* = 0.004) and TCL (*P* = 0.001) groups (Table 2). TFL could decrease uric acid in both men (*P* = 0.036) and women (*P* = 0.041); while TCL decreased uric acid in men (*P* < 0.001) (Tables 3 and 4).

**Figure 1:**
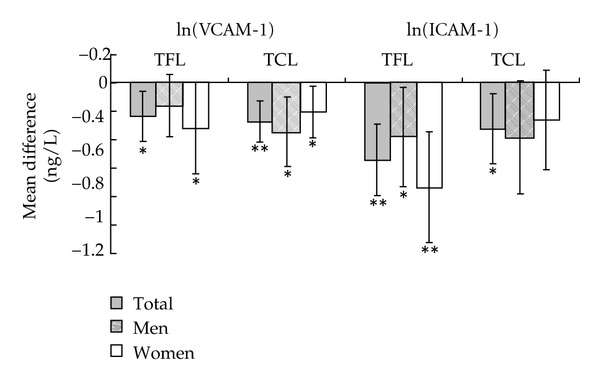
Mean differences of serum intercellular adhesion molecule-1 (ICAM-1) and vascular cell adhesion molecule-1 (VCAM-1) before and after the intervention (ln transformed). Values are means and 95% CIs. *P < 0.05, compared with baseline; **P < 0.01, compared with baseline. ICAM-1, intercellular adhesion molecule-1; VCAM-1, vascular cell adhesion molecule-1; TCL, traditional Chinese liquor; TFL, tea-flavor liquor.

Serum ICAM-1 and VCAM-1 were significantly decreased in TFL (*P* < 0.001 for ICAM-1, *P* = 0.012 for VCAM-1) and TCL (*P* = 0.013 for ICAM-1, *P* = 0.001 for VCAM-1) groups (Figure 1). Men and women in TFL and TCL group had similar response for ICAM-1 and VCAM-1 changes.

### 3.6. Changes in Liver and Kidney Function Parameters, Whole blood Platelet Aggregation, and Other Whole Blood Parameters in Young Subjects

All the liver, kidney function parameters and whole blood parameters were in normal levels and no significant changes took place in these parameters (data not shown).

**Figure 2:**
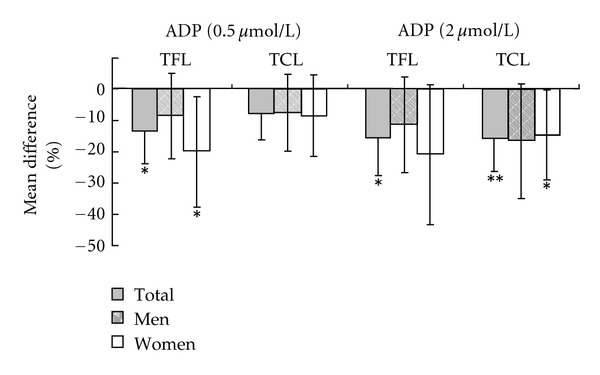
Mean differences of 0.5 *μ*mol/L and 2 *μ*mol/L adenosine-diphosphate- (ADP-) induced platelet aggregation before and after the intervention. Values are means and 95% CIs. *P < 0.05, compared with baseline; **P < 0.01, compared with baseline. TCL, traditional Chinese liquor; TFL, tea-flavor liquor.

Both 0.5 *μ*mol/L (*P* = 0.012) and 2.0 *μ*mol/L (*P* = 0.015) ADP-induced platelet aggregation was remarkably decreased after TFL consumption; TCL could significantly decrease 2.0 *μ*mol/L ADP-induced platelet aggregation (*P* = 0.007), but marginally decrease 0.5 *μ*mol/L ADP-induced platelet aggregation (*P* = 0.056). Stratified analyses indicated that women tended to be more sensitive than men in the change of ADP-induced platelet aggregation (Figure 2). No significant changes of epinephrine-induced platelet aggregation (2.78 *μ*mol/L, 5.56 *μ*mol/L) were observed in TFL and TCL groups.

## 4. Discussion

To our knowledge, this was the first randomized controlled trial conducted to examine the effects of Chinese liquor consumption on CVD risk factors in humans. Approximately one drink of alcohol (13.5 g ethanol) per day was used in this study; and it was consistent with the general alcohol consumption recommendation [10]. To examine the different response, to Chinese liquors between men and women, all the subjects were allocated to consume the same amount of ethanol. The results indicated that both Chinese liquors could protect CVD risk factors, but there were no significant differences between the two liquors. Female subjects appeared to be more sensitive to alcohol than males in relation to serum lipids and platelet aggregation.

There have been numerous epidemiological studies demonstrating the beneficial effects of alcohol consumption on serum lipids levels, including HDL-C [11], TC/HDL-C [12], and other lipid profiles [1]. Our results were consistent with previous studies [1, 10, 12] and showed that Chinese liquor consumption (both TFL and TCL) could improve serum lipid profiles in young healthy subjects, especially for female subjects. In addition, although excess alcohol drinking could cause an increase in blood pressure [13], TFL consumption tended to decrease SBP and this was consistent with Gillman et al. [13] that the lowest SBP occurred in subjects having 1 to <2 drinks per day and a J-shaped curve was indicated by the authors. In contrast, TFL caused a significant increase of DBP, mainly in females and this may due to that females are more sensitive to alcohol exposure [11]. However, DBP of TFL-females were all in normal range (60–76 mmHg) at day 28, which would not possibly cause negative effects.

Alcohol exerted its protective effects on CVD not only by improving blood lipids, but also by decreasing platelet aggregation and endothelial adhesion molecules [5, 9, 14, 15]. The relationship of platelet aggregation with CVD risk has been well established [16]. Consumption of TFL and TCL in our study could significantly decrease 0.5 *μ*mol/L and 2.0 *μ*mol/L ADP-induced platelet aggregation which indicated an important protective effect of both TFL and on CVD in humans. Circulating endothelial adhesion molecules, VCAM-1 and ICAM-1, were supposed to be early markers of atherosclerosis [17]. The decreases of VCAM-1 and ICAM-1 of the present study suggested that moderate consumption of TFL and TCL may have a protective effect on the initial phases of the atherosclerosis process.

Fasting insulin and glucose were reported to be positively related to CVD risk [18]. Fasting serum glucose was significantly decreased in all the groups in the present study, which might represent protective effect of the liquors on CVD, and this might be due to the enhanced insulin secretion and increase of hepatic cytosolic NADH-to-NAD^+^ ratio which inhibits gluconeogenesis [19]. The glucose lowering effect was consistent with Shai et al. [20]; during a 3-month randomized controlled trial, 150 mL of wine consumption significantly decreased fasting plasma glucose among patients with type 2 diabetes. However, there was also well-designed clinical trial demonstrating that alcohol consumption did not affect fasting glucose in postmenopausal women [21]. So the results are still inconsistent. In addition, alcohol consumption was reported to increase insulin sensitivity [21], but neither TFL nor TCL exerted any significant effect on HOMA scores in the present study.

Fasting serum uric acid was an independent risk factor for CVD [22], and alcohol was reported to be related with higher serum uric acid level [23]; and this relationship was more apparent for beer consumption because of its purine content [23]. A recent prospective study [24] found that habitual alcohol intake remarkably contributed to the development of hyperuricaemia, regardless of alcoholic beverage types. Further, ethanol consumption has long been related to hyperuricemia by enhancing the adenine nucleotides degradation and increases lactic acid level in blood [25]. However, our findings indicated that Chinese liquor consumption could decrease the fasting serum uric acid in healthy young men and women, which was quite unexpected. It has been reported that in heavy alcohol drinkers, concentrations of serum uric acid were reduced due to loss of hepatic xanthine oxidase activity [26]. But the amount of alcohol drinking in the present study was rather limited and the results still seemed puzzling. We hypothesized that the nonalcoholic components of the liquors might contribute to the decreased uric acid levels. This was the first randomized trial demonstrating that moderate Chinese liquor intake could decrease serum uric acid, and the mechanisms were yet to be demonstrated.

Many studies reported that alcohol per se protected people from CVD risk [6, 27], while others also indicated the beneficial effects of nonalcoholic components, especially bioflavonoids in red wine [28]. So far, no studies have investigated the potential impact of nonalcoholic components on the protective effects of Chinese liquor on CVD risk factors. Esters and organic acids were major nonalcoholic components in Chinese liquors, and the synergistic influences of these components combined with ethanol on CVD risk factors were yet to be discovered. We chose two Chinese liquors containing the same ethanol content but with different total ester and organic acid levels. However there were no significant differences between the effects of TFL and TCL on CVD risk factors. In addition, there might be synergistic effects of ethanol and nonalcoholic components in Chinese liquors on the CVD risk factors, such as the decrease of uric acid, which has not been observed previously, and the mechanism warranted further investigation.

There are some limitations in the present study. Firstly, sample size in each group was small and study period is short. However, we recruited both male and female subjects to assess the protective effects of Chinese liquor on CVD, which would make the results more persuasive; and young healthy subjects were chosen to avoid unpredictable bias caused by potential health problems. Secondly, subjects were asked to follow their regular physical activity, diet habits, and overall lifestyle, and during the period, incompliance would possibly happen; however, diet records did not find significant change of diet habits. Alcohol consumption compliance within the included subjects was 100% as all the subjects come to a certain room to drink. Finally, as we intended to compare the effects of the two Chinese liquors, we did not include a control group, which was a major limitation in the present study.

In conclusion, consumption of both Chinese liquors, TFL and TCL, exerted protective effects on CVD risk factors in relation to serum lipids, whole blood platelet aggregation, endothelial adhesion molecules (VCAM-1, ICAM-1), uric acid and serum glucose. No significant differences were observed between the effects of the two Chinese liquors. However, the results were valid only for 28 days, and the possibility of adverse effect (liver, kidney) of chronic alcohol consumption should be considered.

## Figures and Tables

**Table 1:** Baseline characteristics and dietary nutrient intake of subjects included in TFL and TCL groups.

Variables	TFL			TCL		
	Total (*n* = 23)	Men (*n* = 12)	Women (*n* = 11)	Total (*n* = 22)	Men (*n* = 11)	Women (*n* = 11)
*Subject characteristics*						
Age (year)	23.6 ± 1.5	23.7 ± 1.8	23.5 ± 1.13	23.6 ± 1.4	22.9 ± 0.3	24.3 ± 1.7
BMI (kg/m^2^)	21.3 ± 1.6	21.6 ± 1.5	21.0 ± 1.8	21.1 ± 2.2	21.5 ± 2.0	20.6 ± 2.4
Body weight (kg)	58.8 ± 7.3	63.3 ± 5.2	54.0 ± 6.1	59.0 ± 9.3	65.0 ± 6.7	53.0 ± 7.6
TG (mmol/L)	0.9 ± 0.3	1.0 ± 0.4	0.8 ± 0.2	0.9 ± 0.3	0.8 ± 0.2	0.9 ± 0.4
TC (mmol/L)	4.2 ± 0.8	4.2 ± 0.9	4.2 ± 0.6	4.4 ± 0.7	4.5 ± 0.7	4.3 ± 0.6
HDL-C (mmol/L)	1.4 ± 0.2	1.3 ± 0.2	1.4 ± 0.2	1.5 ± 0.4	1.5 ± 0.3	1.5 ± 0.4
LDL-C (mmol/L)	2.2 ± 0.7	2.2 ± 0.8	2.1 ± 0.6	2.3 ± 0.5	2.4 ± 0.7	2.2 ± 0.4
SBP (mmHg)	110.6 ± 13.2	119.7 ± 10.5	100.7 ± 7.6	108.8 ± 10.2	114.4 ± 11.4	103.2 ± 4.4
DBP (mmHg)	68.5 ± 9.8	73.5 ± 10.0	62.2 ± 6.4	68.4 ± 6.4	70.9 ± 7.3	65.8 ± 4.3
Glucose (mmol/L)	5.1 ± 0.4	5.2 ± 0.2	5.0 ± 0.5	5.0 ± 0.3	5.1 ± 0.3	5.0 ± 0.3
*Dietary nutrient intake*						
Total energy (kcal)	2056 ± 321	2282 ± 238	1810 ± 192	1997 ± 282	2160 ± 282	1834 ± 171
Carbohydrate (g)	299 ± 81	330 ± 73	265 ± 78	313 ± 78	344 ± 80	283 ± 66
Total fat (g)	65.4 ± 25.2	72.9 ± 25.5	57.3 ± 23.3	53.9 ± 21.6	56.1 ± 19.3	51.8 ± 24.4
Protein (g)	73.1 ± 14.5	81.1 ± 13.6	64.3 ± 9.7	69.9 ± 14.2	75.1 ± 12.4	64.7 ± 14.5
Carbohydrate (% of energy )	57.9 ± 12.1	57.8 ± 10.8	58.0 ± 13.9	62.0 ± 10.3	63.2 ± 8.5	60.1 ± 12.1
Fat (% of energy )	28.9 ± 11.1	28.8 ± 10.1	28.9 ± 12.7	25.0 ± 10.1	23.7 ± 8.1	27.0 ± 11.7
Protein (% of energy)	14.3 ± 2.4	14.3 ± 2.4	14.3 ± 2.6	14.0 ± 3.3	14.0 ± 2.6	14.3 ± 4.3

The values were expressed as mean ± SD.

TFL: tea-flavor liquor; TCL: traditional Chinese liquor; TG: triacylglycerol; TC: total cholesterol; HDL-C: high-density lipoprotein cholesterol; LDL-C: low-density lipoprotein cholesterol; BMI: body mass index; DBP: diastolic blood pressure; SBP: systolic blood pressure.

**Table 2:** Fasting concentrations of cardiovascular disease risk factors before and after the intervention in all subjects.

Variables	TFL (*n* = 23)			TCL (*n* = 22)			*P* value		
	Day 0	Day 28	Difference (95% CIs)	Day 0	Day 28	Difference (95% CIs)	Time	Liquor	Time × Liquor
Body weight (kg)	58.80 ± 7.29	58.59 ± 7.45	−0.22 (−0.92, 0.48)	59.02 ± 9.29	58.73 ± 9.06	−0.30 (−0.86, 0.27)	0.786	0.825	0.871
HDL-C (mmol/L)	1.37 ± 0.19	1.43 ± 0.26	0.06 (−0.01, 0.12)	1.49 ± 0.36	1.53 ± 0.38	0.04 (−0.04, 0.12)	0.001	0.224	0.562
LDL-C (mmol/L)	2.17 ± 0.69	2.11 ± 0.65	−0.06 (−0.18, 0.06)	2.27 ± 0.53	2.10 ± 0.45	−0.17 (−0.32, −0.01)^∗^	0.002	0.808	0.218
TC (mmol/L)	4.17 ± 0.77	3.90 ± 0.68	−0.27 (−0.47, −0.07)^∗^	4.41 ± 0.67	4.04 ± 0.63	−0.37 (−0.58, −0.16)^∗∗^	<0.001	0.338	0.403
HDL-C/LDL-C	0.71 ± 0.33	0.76 ± 0.36	0.05 (0.02, 0.08)^∗∗^	0.68 ± 0.18	0.74 ± 0.18	0.06 (0.02, 0.10)^∗∗^	0.106	0.748	0.635
TC/HDL-C	3.09 ± 0.65	2.79 ± 0.64	−0.29 (−0.36, −0.22)^∗∗^	3.07 ± 0.63	2.74 ± 0.53	−0.33 (−0.42, −0.24)^∗∗^	0.017	0.853	0.53
TG (mmol/L)	0.91 ± 0.34	0.76 ± 0.24	−0.15 (−0.23, −0.07)^∗∗^	0.85 ± 0.32	0.80 ± 0.37	−0.06 (−0.18, 0.06)	0.953	0.955	0.176
Apo A1 (g/L)	1.37 ± 0.15	1.48 ± 0.20	0.11 (0.04, 0.18)^∗∗^	1.45 ± 0.29	1.57 ± 0.29	0.12 (0.05, 0.19)^∗∗^	0.515	0.207	0.815
Apo B (g/L)	0.50 ± 0.13	0.51 ± 0.11	0.01 (−0.03, 0.05)	0.51 ± 0.08	0.50 ± 0.11	−0.01 (−0.05, 0.03)	0.716	0.877	0.401
Apo B/A1	0.37 ± 0.10	0.35 ± 0.09	−0.02 (−0.04, 0.01)	0.37 ± 0.10	0.33 ± 0.09	−0.04 (−0.06, −0.01)^∗∗^	0.702	0.739	0.224
DBP (mmHg)	68.27 ± 10.3	73.05 ± 7.47	5.04 (2.19, 7.90)^∗∗^	68.36 ± 6.4	69.91 ± 8.76	1.55 (−1.40, 4.50)	0.094	0.487	0.089
SBP (mmHg)	110.61 ± 13.21	106.13 ± 9.76	−4.48 (−8.23, −0.73)^∗^	108.77 ± 10.2	104.68 ± 10.7	−4.09 (−8.76, 0.58)	0.053	0.534	0.912
Glucose (mmol/L)	5.10 ± 0.39	4.75 ± 0.34	−0.35 (−0.50, −0.20)^∗∗^	5.02 ± 0.28	4.64 ± 0.45	−0.38 (−0.56, −0.21)^∗∗^	0.287	0.293	0.759
Insulin (*μ*IU/mL)	4.81 ± 1.69	5.42 ± 1.80	0.61 (−0.36, 1.59)	4.0 ± 1.95	5.34 ± 2.05	1.34 (0.26, 2.43)^∗^	0.104	0.303	0.297
HOMA	1.10 ± 0.45	1.15 ± 0.39	0.05 (−0.19, 0.28)	0.90 ± 0.45	1.12 ± 0.49	0.22 (−0.03, 0.47)	0.149	0.282	0.293
Uric acid (*μ*mol/L)	344.64 ± 93.11	314.14 ± 71.38	−30.49 (−50.33, −10.65)^∗∗^	332.78 ± 75	290.41 ± 51.7	−42.36 (−65.65, −19.07)^∗∗^	0.001	0.235	0.388
Hs-CRP (mg/L)	0.75 ± 0.47	1.04 ± 0.49	0.30 (−0.02, 0.62)	0.69 ± 0.60	0.81 ± 0.48	0.12 (−0.11, 0.36)	0.782	0.199	0.375

TFL: tea-flavor liquor; TCL: traditional Chinese liquor; TG: triacylglycerol; TC: total cholesterol; HDL-C: high-density lipoprotein cholesterol; LDL-C: low-density lipoprotein cholesterol; ApoA1: apolipoprotein A1; ApoB: apolipoprotein B; DBP: diastolic blood pressure; SBP: systolic blood pressure; HOMA: Homeostasis Model Assessment. *P < 0.05, compared to day 0 for each intervention; **P < 0.01, compared to day 0 for each intervention.

**Table 3:** Fasting concentrations of cardiovascular disease risk factors before and after the intervention in female subjects.

Variables	TFL-female (*n* = 11)			TCL-female (*n* = 11)			*P* value		
	Day 0	Day 28	Difference (95% CIs)	Day 0	Day 28	Difference (95% CIs)	Time	Liquor	Time × Liquor
Body weight (kg)	53.96 ± 6.08	53.41 ± 6.09	−0.55 (−1.61, 0.52)	53 ± 7.57	52.73 ± 7.15	−0.27 (−1.23, 0.68)	0.217	0.778	0.676
HDL (mmol/L)	1.43 ± 0.15	1.59 ± 0.15	0.16 (0.09, 0.24)^∗∗^	1.48 ± 0.40	1.61 ± 0.46	0.12 (0, 0.24)^∗^	<0.001	0.504	0.817
LDL-C (mmol/L)	2.11 ± 0.58	2.18 ± 0.59	0.07 (−0.04, 0.19)	2.16 ± 0.38	2.11 ± 0.31	−0.05 (−0.34, 0.23)	0.892	0.93	0.939
TC (mmol/L)	4.16 ± 0.62	4.14 ± 0.58	−0.02 (−0.21, 0.17)	4.34 ± 0.64	4.12 ± 0.68	−0.22 (−0.60, 0.16)	0.225	0.732	0.309
HDL-C/LDL-C	0.74 ± 0.29	0.79 ± 0.29	0.05 (0.01, 0.09)^∗^	0.69 ± 0.17	0.75 ± 0.15	0.06 (−0.01, 0.13)	0.005	0.669	0.814
TC/HDL-C	2.91 ± 0.35	2.60 ± 0.29	−0.32 (−0.41, −0.22)^∗∗^	3.05 ± 0.64	2.68 ± 0.50	−0.37 (−0.53, −0.22)^∗∗^	<0.001	0.554	0.501
TG (mmol/L)	0.79 ± 0.18	0.64 ± 0.14	−0.14 (−0.23, −0.05)^∗∗^	0.92 ± 0.41	0.78 ± 0.34	−0.14 (−0.30, 0.02)	0.003	0.265	0.97
Apo A1 (g/L)	1.42 ± 0.15	1.61 ± 0.15	0.20 (0.10, 0.29)^∗∗^	1.50 ± 0.33	1.65 ± 0.35	0.15 (0.02, 0.27)^∗^	<0.001	0.592	0.491
Apo B (g/L)	0.48 ± 0.10	0.50 ± 0.11	0.02 (−0.01, 0.05)	0.50 ± 0.06	0.49 ± 0.04	−0.01 (−0.06, 0.04)	0.575	0.889	0.24
Apo B/A1	0.34 ± 0.07	0.31 ± 0.05	−0.03 (−0.05, −0.01)^∗∗^	0.35 ± 0.08	0.31 ± 0.07	−0.03 (−0.06, −0.01)^∗^	0.001	0.939	0.806
DBP (mmHg)	62.18 ± 6.37	67.91 ± 5.28	5.73 (2.06, 9.40)^∗^	65.82 ± 4.31	65.27 ± 5.90	−0.55 (−3.42, 2.33)	0.022	0.815	0.007
SBP (mmHg)	100.73 ± 7.59	100.64 ± 9.17	−0.09 (−6.26, 6.08)	103.18 ± 4.36	97.91 ± 10.63	−5.27 (−12.14, 1.59)	0.21	0.962	0.225
Glucose (mmol/L)	4.96 ± 0.48	4.60 ± 0.36	−0.36 (−0.60, −0.12)^∗∗^	4.95 ± 0.27	4.45 ± 0.53	−0.50 (−0.76, −0.23)^∗∗^	<0.001	0.634	0.415
Insulin (*μ*IU/mL)	5.15 ± 1.62	6.00 ± 2.06	0.84 (−0.84, 2.52)	4.22 ± 1.51	4.74 ± 1.90	0.52 (−0.77, 1.82)	0.168	0.081	0.741
HOMA	1.16 ± 0.50	1.24 ± 0.45	0.08 (−0.34, 0.49)	0.94 ± 0.37	0.96 ± 0.47	0.03 (−0.28, 0.33)	0.667	0.123	0.829
Uric acid (*μ*mol/L)	273.25 ± 64.80	252.12 ± 48.61	−21.13 (−41.15, −1.10)^∗^	278.39 ± 50.53	254 ± 38.99	−24.39 (−65.10, 16.32)	0.037	0.859	0.874
Hs-CRP (mg/L)	0.57 ± 0.41	0.71 ± 0.58	0.14 (−0.06, 0.34)	0.55 ± 0.73	0.57 ± 0.15	0.02 (−0.41, 0.45)	0.117	0.109	0.145

TFL: tea-flavor liquor; TCL: traditional Chinese liquor; TG: triacylglycerol; TC: total cholesterol; HDL-C: high-density lipoprotein cholesterol; LDL-C: low-density lipoprotein cholesterol; ApoA1: apolipoprotein A1; ApoB: apolipoprotein B; DBP: diastolic blood pressure; SBP: systolic blood pressure; HOMA: Homeostasis Model Assessment. *P < 0.05, compared to day 0 for each intervention; **P < 0.01, compared to day 0 for each intervention.

**Table 4:** Fasting concentrations of cardiovascular disease risk factors before and after the intervention in male subjects.

Variables	TFL-male (*n* = 12)			TCL-male (*n* = 11)			*P* value		
	Day 0	Day 28	Difference (95% CIs)	Day 0	Day 28	Difference (95% CIs)	Time	Liquor	Time × Liquor
Body weight (kg)	63.25 ± 5.25	63.33 ± 5.09	0.08 (−0.97, 1.14)	65.05 ± 6.66	64.73 ± 6.48	−0.32 (−1.10, 0.47)	0.702	0.52	0.514
HDL-C (mmol/L)	1.31 ± 0.21	1.28 ± 0.24	−0.04 (−0.11, 0.04)	1.49 ± 0.33	1.45 ± 0.29	−0.04 (−0.14, 0.05)	0.16	0.118	0.913
LDL-C (mmol/L)	2.23 ± 0.79	2.05 ± 0.72	−0.18 (−0.38, 0.02)	2.37 ± 0.65	2.10 ± 0.58	−0.28 (−0.45, −0.11)^∗∗^	0.001	0.737	0.406
TC (mmol/L)	4.18 ± 0.93	3.68 ± 0.70	−0.50 (−0.82, −0.19)^∗∗^	4.48 ± 0.72	3.96 ± 0.60	−0.52 (−0.71, −0.34)^∗∗^	<0.001	0.353	0.917
HDL-C/LDL-C	0.69 ± 0.36	0.74 ± 0.42	0.05 (0, 0.11)	0.67 ± 0.20	0.74 ± 0.21	0.07 (0.01, 0.12)^∗^	0.003	0.934	0.676
TC/HDL-C	3.25 ± 0.82	2.98 ± 0.82	−0.27 (−0.38, −0.16)^∗∗^	3.08 ± 0.65	2.79 ± 0.58	−0.28 (−0.39, −0.17)^∗∗^	<0.001	0.565	0.86
TG (mmol/L)	1.02 ± 0.42	0.86 ± 0.26	−0.16 (−0.31, −0.02)^∗^	0.79 ± 0.19	0.81 ± 0.42	0.02 (−0.17, 0.21)	0.202	0.299	0.108
Apo A1 (g/L)	1.33 ± 0.14	1.36 ± 0.17	0.03 (−0.06, 0.11)	1.40 ± 0.24	1.50 ± 0.20	0.10 (0, 0.19)^∗^	0.04	0.175	0.252
Apo B (g/L)	0.51 ± 0.16	0.52 ± 0.12	0.003 (−0.07, 0.08)	0.53 ± 0.10	0.51 ± 0.16	−0.01 (−0.08, 0.06)	0.871	0.925	0.761
Apo B/A1	0.39 ± 0.12	0.39 ± 0.10	−0.004 (−0.05, 0.04)	0.39 ± 0.12	0.35 ± 0.11	−0.04 (−0.08, 0.01)	0.132	0.667	0.217
DBP (mmHg)	73.50 ± 9.97	77.92 ± 5.42	4.42 (−0.47, 9.30)	70.91 ± 7.30	74.55 ± 8.89	3.64 (−1.78, 9.06)	0.023	0.323	0.815
SBP (mmHg)	119.67 ± 10.47	111.17 ± 7.51	−8.50 (−12.35, −4.65)^∗∗^	114.36 ± 11.40	111.45 ± 4.93	−2.91 (−10.42, 4.61)	0.006	0.448	0.146
Glucose (mmol/L)	5.23 ± 0.24	4.89 ± 0.25	−0.34 (−0.55, −0.12)^∗∗^	5.09 ± 0.28	4.81 ± 0.26	−0.27 (−0.51, −0.03)^∗^	<0.001	0.188	0.672
Insulin (*μ*IU/mL)	4.50 ± 1.75	4.90 ± 1.41	0.40 (−0.93, 1.73)	3.77 ± 2.36	5.93 ± 2.12	2.16 (0.34, 3.99)^∗^	0.019	0.811	0.094
HOMA	1.04 ± 0.40	1.07 ± 0.32	0.02 (−0.29, 0.34)	0.86 ± 0.54	1.28 ± 0.48	0.42 (0, 0.84)	0.073	0.932	0.108
Uric acid (*μ*mol/L)	410.08 ± 61.27	371 ± 25.87	−39.08 (−75.21, −2.94)^∗^	387.16 ± 52.46	326.83 ± 34.31	−60.34 (−85.58, −35.10)^∗∗^	<0.001	0.051	0.307
Hs-CRP (mg/L)	0.94 ± 0.47	1.03 ± 0.41	0.09 (−0.31, 0.49)	0.83 ± 0.42	1.06 ± 0.57	0.23 (−0.04, 0.50)	0.144	0.817	0.527

TFL: tea-flavor liquor; TCL: traditional Chinese liquor; TG: triacylglycerol; TC: total cholesterol; HDL-C: high-density lipoprotein cholesterol; LDL-C: low-density lipoprotein cholesterol; ApoA1: apolipoprotein A1; ApoB: apolipoprotein B; DBP: diastolic blood pressure; SBP: systolic blood pressure; HOMA: Homeostasis Model Assessment. *P < 0.05, compared to day 0 for each intervention; **P < 0.01, compared to day 0 for each intervention.

## Abbreviations

CVD: Cardiovascular disease
TCL: Traditional Chinese liquor
TFL: Tea-flavor liquor
TC: Total cholesterol
TG: Triacylglycerol
HDL-C: High-density lipoprotein cholesterol
LDL-C: Low-density lipoprotein cholesterol
hs-CRP: High-sensitivity C-reactive protein
ADP: Adenosine diphosphate
apoA1/B: ApolipoproteinA1/B
HOMA: Homeostasis Model Assessment
ICAM-1: Intercellular adhesion molecule-1
VCAM-1: Vascular cell adhesion molecule-1
DBP: Diastolic blood pressure
SBP: Systolic blood pressure.
